# The flaxseed lignan secoisolariciresinol diglucoside decreases local inflammation, suppresses NFκB signaling, and inhibits mammary tumor growth

**DOI:** 10.1007/s10549-018-5021-6

**Published:** 2018-10-26

**Authors:** Laura W. Bowers, Claire G. Lineberger, Nikki A. Ford, Emily L. Rossi, Arunima Punjala, Kristina K. Camp, Bruce K. Kimler, Carol J. Fabian, Stephen D. Hursting

**Affiliations:** 1 Department of Nutrition, University of North Carolina, 135 Dauer Drive, CB #7461, Chapel Hill, NC 27599 USA; 2 Lineberger Comprehensive Cancer Center, University of North Carolina, 450 West Drive, Chapel Hill, NC 27514 USA; 3 Department of Nutritional Sciences, University of Texas at Austin, 1400 Barbara Jordan Blvd, R1800, Austin, TX 78723 USA; 4 Department of Radiation Oncology, University of Kansas Medical Center, 3901 Rainbow Boulevard, Kansas City, KS 66160 USA; 5 Department of Internal Medicine, University of Kansas Medical Center, 3901 Rainbow Boulevard, Kansas City, KS 66160 USA; 6 Nutrition Research Institute, University of North Carolina, 500 Laureate Way, Kannapolis, NC 28081 USA

**Keywords:** Breast cancer, Secoisolariciresinol diglucoside (SDG), Plant lignan, Enterolactone (ENL), Enterodiol (END), NFκB

## Abstract

**Purpose:**

Exposure to the polyphenolic plant lignan secoisolariciresinol diglucoside (SDG) and its metabolite enterolactone (ENL) has been associated with reduced breast cancer progression, particularly for estrogen receptor alpha (ERα)-negative disease, and decreased preclinical mammary tumor growth. However, while preclinical studies have established that SDG and ENL affect measures of progression in models of triple-negative breast cancer (TNBC, a subset of ERα-negative disease), the molecular mechanisms underlying these effects remain unclear.

**Methods:**

C57BL/6 mice were fed a control diet (control, 10% kcal from fat) or control diet + SDG (SDG, 100 mg/kg diet) for 8 weeks, then orthotopically injected with syngeneic E0771 mammary tumor cells (a model of TNBC); tumor growth was monitored for 3 weeks. The role of reduced NF-κB signaling in SDG’s anti-tumor effects was explored in vitro via treatment with the bioactive SDG metabolite ENL. In addition to the murine E0771 cells, the in vitro studies utilized MDA-MB-231 and MCF-7 cells, two human cell lines which model the triple-negative and luminal A breast cancer subtypes, respectively.

**Results:**

SDG supplementation in the mice significantly reduced tumor volume and expression of phospho-p65 and NF-κB target genes (*P* < 0.05). Markers of macrophage infiltration were decreased in the distal-to-tumor mammary fat pad of mice supplemented with SDG relative to control mice (*P* < 0.05). In vitro, ENL treatment inhibited viability, survival, and NF-κB activity and target gene expression in E0771, MDA-MB-231, and MCF-7 cells (*P* < 0.05). Overexpression of *Rela* attenuated ENL’s inhibition of E0771 cell viability and survival.

**Conclusions:**

SDG reduces tumor growth in the E0771 model of TNBC, likely via a mechanism involving inhibition of NF-κB activity. SDG could serve as a practical and effective adjuvant treatment to reduce recurrence, but greater understanding of its effects is needed to inform the development of more targeted recommendations for its use.

**Electronic supplementary material:**

The online version of this article (10.1007/s10549-018-5021-6) contains supplementary material, which is available to authorized users.

## Background

Secoisolariciresinol diglucoside (SDG) is a polyphenolic plant lignan found in flaxseeds and other oil-rich seeds and nuts as well as legumes, whole grains, certain fruits and vegetables, coffee, tea, and wine [1, 2]. Following oral consumption, SDG is hydrolyzed to secoisolariciresinol and then metabolized by intestinal bacteria to two biologically active enterolignans that have been classified as phytoestrogens: enterolactone (ENL) and enterodiol (END) [3]. Studies have generally found significant inverse associations between lignan exposure and breast cancer mortality [4–8]. However, only one study included premenopausal women [6] and another found that effects were limited to estrogen receptor alpha (ERα)-negative tumors [4]. Consequently, it is uncertain whether all breast cancer patients would benefit from greater SDG intake post-diagnosis.

To further explore SDG’s impact on breast cancer, several preclinical studies have examined the effects of lignan exposure on animal models of both pre- and postmenopausal ERα-positive breast cancer, with the vast majority demonstrating significant reductions in mammary tumor growth or preneoplastic changes [9–18]. These anti-tumor effects have been linked to decreased proliferation and angiogenesis as well as increased apoptosis [9–11, 13, 15–17]. However, only a small number of studies have investigated the possible molecular pathways underlying the anticancer effects of SDG [10, 19–21]. In addition, there has been relatively little exploration of SDG’s effects on models of triple-negative breast cancer (TNBC), despite epidemiologic data suggesting enterolignans may have a stronger protective effect against mortality from ERα-negative tumors [4]. Researchers have demonstrated that SDG metabolites reduce proliferation, adhesion, migration, and invasion in the triple-negative MDA-MB-231 breast cancer cell line and increase these cells’ response to radiation and chemotherapy [22–25], but the molecular pathways responsible for these effects have not been established.

The current study examined the impact of SDG supplementation on in vivo growth of orthotopically injected E0771 mouse mammary tumor cells, a syngeneic model of basal-like TNBC [21, 26]. After demonstrating that SDG inhibits E0771 tumor growth in association with decreased tumor activity of the inflammation-regulating transcription factor nuclear factor-kappa B (NF-κB), we explored connections between these factors using in vitro models of multiple breast cancer subtypes. Greater understanding of SDG’s effects on different breast cancer models and the mechanisms mediating these effects will inform the development of more targeted recommendations regarding the use of SDG supplementation for reducing the burden of breast cancer.

## Methods

### In vivo dose-finding pilot study

Animal studies and procedures were approved and monitored by the University of Texas Institutional Animal Care and Use Committee. Female, 10-week-old C57BL/6 mice were purchased from Charles River Laboratories, Inc., and fed a control diet (10% kcal from fat, catalog #D12450J, Research Diets, Inc.) ad libitum for 8 weeks. Mice were then randomized to the control diet (*n* = 10) or 1 of 2 control + SDG diets (low-dose SDG: 25 mg/kg diet, *n* = 10; high-dose SDG: 74 mg/kg diet, *n* = 10) for 8 weeks. The goal of this study was to establish the concentration of SDG that would result in serum ENL and END levels comparable to those achieved in a 12-month pilot clinical trial of SDG supplementation in women [27]. SDG for both in vivo studies was obtained from Barleans Organic Oils, LLC (Ferndale, WA, USA). Following euthanization, blood was collected by cardiac puncture and serum stored at − 80 °C. The 4th mammary gland was excised, flash-frozen in liquid nitrogen and stored at − 80 °C.

### In vivo tumor study

Animal studies and procedures were approved and monitored by the University of North Carolina Institutional Animal Care and Use Committee. Female, 12-week-old C57BL/6 mice were purchased from Charles River Laboratories, Inc., and fed the control diet ad libitum for 2 weeks. Mice were then randomized to the control diet (*n* = 20) or control + SDG diet (100 mg SDG/kg diet, *n* = 20). This dose was chosen because the serum ENL and END levels in the high-dose (74 mg/kg diet) pilot group were ~ 25% lower than in the clinical trial subjects [27]. After 7 weeks, blood was collected from all mice by submandibular bleed, and serum stored at − 80 °C. One week later, all mice were orthotopically injected with 3.5 × 10^4^ syngeneic E0771 mammary tumor cells. In vivo tumor growth was measured twice/week with digital calipers, and all mice euthanized 3 weeks after injection. Mice remained on their respective control or control + SDG diets throughout the study. Tumors and the 4th and 9th mammary glands were excised and divided to be formalin fixed and paraffin-embedded or flash-frozen in liquid nitrogen and stored at − 80 °C. The ellipsoid equation was used to determine volume ex vivo: 1/6π(D1 × D2 × D3). Body fat was assessed after euthanization using a Lunar PIXImus Dual Emission X-Ray Absorptiometer (GE Medical Systems, Ontario, CA).

### Serum enterolactone and cytokine measurement

To measure serum ENL and END concentrations, samples underwent solid-phase extraction and overnight enzymatic hydrolysis. The unconjugated lignans were then isolated by solid-phase extraction, converted to *tert*-butyldimethylsilyl ethers, and analyzed by gas chromatography mass spectrometry [28]. Serum cytokines were analyzed by Bio-Plex Multiplex Immunoassay on a Bio-Plex^Ⓡ^ Magpix Multiplex Reader (Bio-Rad, Inc., Hercules, CA, USA).

### Quantitative RT-PCR analyses

Total RNA isolated from tissues and cell culture samples was reverse transcribed and samples assayed in triplicate for individual genes as previously described [29]. Tumor expression of NF-κB target genes was assessed using a Mouse NF-κB Signaling Target RT^2^ Profiler PCR Array (Qiagen, Germantown, MD, USA). All quantitative RT-PCR assays were analyzed using a ViiA™7 RT-PCR System (Applied Biosystems, Waltham, MA, USA).

### Crown-like structure analysis

Paraffin-embedded distal-to-tumor mammary gland tissue (*n* = 6/group) was cut into 4-µm-thick sections and stained with hematoxylin and eosin. The total number of crown-like structures (CLS) per section was quantified, and the mammary tissue area determined using Aperio ImageScope (Leica Biosystems, Buffalo Grove, IL, USA). Prevalence of CLS was quantified as CLS per cm^2^ of mammary tissue.

### Immunohistochemical analyses

Paraffin-embedded tumor tissue (*n* = 6/group) was cut into 4-µm-thick sections and stained, processed and analyzed as previously described [30] with the following primary antibodies: F4/80 (Abcam #ab6640), phospho (p)-p65 (Ser276) (Santa Cruz #sc-101749), and p-STAT3 (Tyr705) (Cell Signaling #9131).

### Cell lines and reagents

One mouse mammary tumor cell line, E0771, and two human breast cancer cell lines, MDA-MB-231 and MCF-7, were used for the in vitro studies. All cell lines were maintained in RPMI 1640 media (GIBCO Life Technologies, Grand Island, NY, USA) supplemented with 10% fetal bovine serum, 10 mM HEPES buffer, and 2 mM l-glutamine (complete media). ENL was purchased from Sigma-Aldrich (St. Louis, MO, USA) and dissolved in ethanol. Two ENL concentrations, 1 µM and 10 µM, were chosen based on the literature [22, 31, 32] and utilized for all in vitro experiments.

### In vitro cell viability and survival assays

For the cell viability assay, cells were seeded at a density of 5 × 10^3^ in 96-well plates. After 24 h, the cells were treated with vehicle, 1 µM or 10 µM ENL in complete media for 48 h. MTT reagent was used to assess cell viability levels as previously described [33, 34]. For the cell survival assay, cells were seeded at a density of 1 × 10^3^ in 6-well plates. After 24 h, the cells were continuously exposed to vehicle, 1 µM or 10 µM ENL in complete media for 7 days, with the treatments replenished on day 4. The colonies were fixed and stained with 0.5% crystal violet in 50% methanol, counted, and imaged with a digital camera on day 7.

### In vitro NF-κB activity

The impact of ENL on NF-κB activity was assessed via a NF-κB Cignal Reporter Assay (Qiagen) and quantitative RT-PCR for NF-κB target gene expression. For the NF-κB Reporter Assay, cells were seeded at a density of 2.5 × 10^3^ in 96-well plates. After 24 h, the cells were transfected with the NF-κB Reporter mixture (an inducible NF-κB-responsive firefly luciferase reporter + a constitutively expressing *Renilla* construct) using FuGENE^Ⓡ^ 6 (Promega, Madison, WI, USA). After another 24-h incubation, cells were treated with vehicle, 1 µM or 10 µM ENL in complete media for 48 h, followed by the same treatments plus LPS (10 ng/ml) in complete media for 24 h. The cells’ luciferase activity was measured using Promega’s Dual Luciferase^Ⓡ^ Reporter Assay System on a Cytation 3 Cell Imaging Multi-Mode Reader (BioTek Instruments Inc.). For NF-κB target gene measurement, cells were seeded at a density of 1.5 × 10^5^ in 6-well plates. After 24 h, cells were treated with vehicle, 1 µM or 10 µM ENL in complete media for 48 h, followed by the same treatments plus LPS (10 ng/ml) in complete media for 24 h. Four NF-κB target genes were chosen to assess in vitro from the 41 target genes with significantly lower expression in SDG mice versus controls; criteria for inclusion were a known link to breast cancer progression and *P* < 0.01 for the in vivo NF-κB target gene array.

### *Rela* overexpression

The Mouse pCMV3-GFPSpark-mRela Plasmid (Rela; a *Rela* overexpression plasmid) and the pCMV3-N-GFPSpark Control Vector (NC), purchased from Sino Biological, Inc. (Beijing, China), were transiently transfected into E0771 cells using FuGENE^Ⓡ^ 6 (Promega). Nuclear p65 expression was measured 48 h after transfection by western blot analysis using NF-κB p65 (D14E12) XP^®^ Rabbit antibody (Cell Signaling #8242). E0771 cells were seeded for the MTT and colony formation assays 48 h after transfection with the Rela and NC plasmids. The assays then proceeded as described above.

### Statistical analyses

Animal study data are presented as mean ± SD and in vitro data as mean ± SEM. All in vitro data shown represent the average of at least 3 independent experiments. For all statistical tests, GraphPad Prism software was used (GraphPad Software Inc., La Jolla, CA, USA). Differences between animals or cells exposed to 2 experimental conditions were analyzed using Student’s *t* test. Differences between cells exposed to more than 2 experimental conditions were analyzed using one-way ANOVA (1 independent variable) or two-way ANOVA (> 1 independent variable), both followed by Tukey’s post hoc test. *P* < 0.05 was considered significantly different.

## Results

### High-dose SDG supplementation increases serum ENL and END levels

**Fig. 1:**
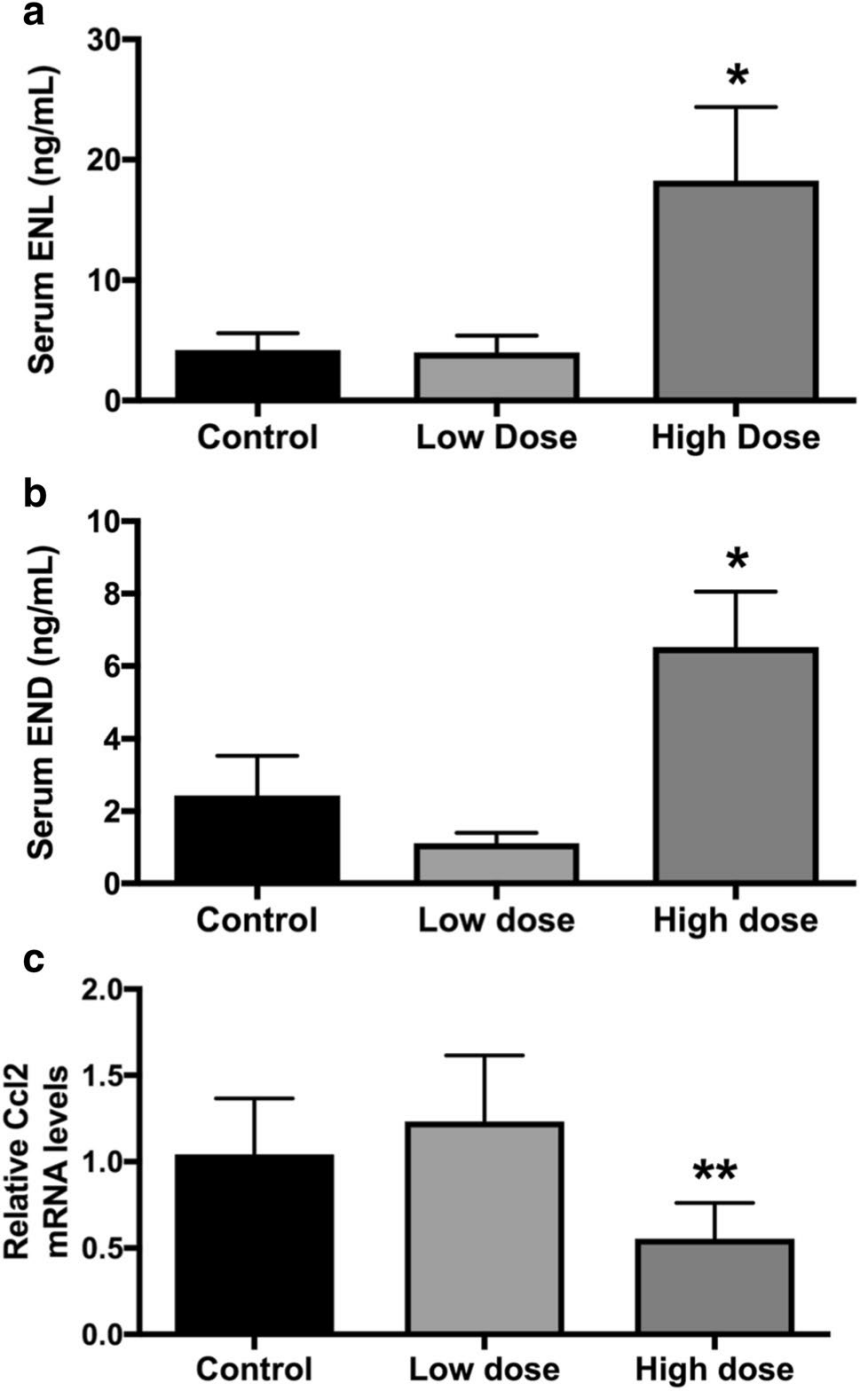
Serum ENL and END levels are increased in mice receiving high-dose SDG. Serum ENL (**a**) and END (**b**) levels were measured in mice receiving a control diet, low-dose SDG-supplemented diet (low dose; 25 mg/kg diet), or high-dose SDG-supplemented diet (high dose; 74 mg/kg diet). **c***Ccl2* gene expression in the 4th mammary gland of control, low-dose, and high-dose mice was measured by quantitative RT-PCR. *P < 0.05; **P < 0.01 in comparison with control

We first performed a pilot animal study to determine the SDG concentration in murine diet that achieves ENL and END levels comparable to those in women that received 50 mg/day of SDG for 12 months in a pilot clinical trial [27]. C57BL/6 mice fed the high-dose diet (74 mg/kg SDG) had significantly greater serum ENL and END levels in comparison with mice fed the control diet (*P* < 0.05 for both) (Fig. 1a, b), though these levels were approximately 25% lower than those achieved in the pilot clinical trial [27]. In addition, mammary tissue gene expression of the pro-inflammatory chemokine *Ccl2* was reduced in mice fed the high-dose SDG diet versus control mice (*P* < 0.001) (Fig. 1c). No significant differences were observed in expression of *Il6* or *Tnf* (data not shown).

### SDG supplementation reduces mammary inflammation

**Fig. 2:**
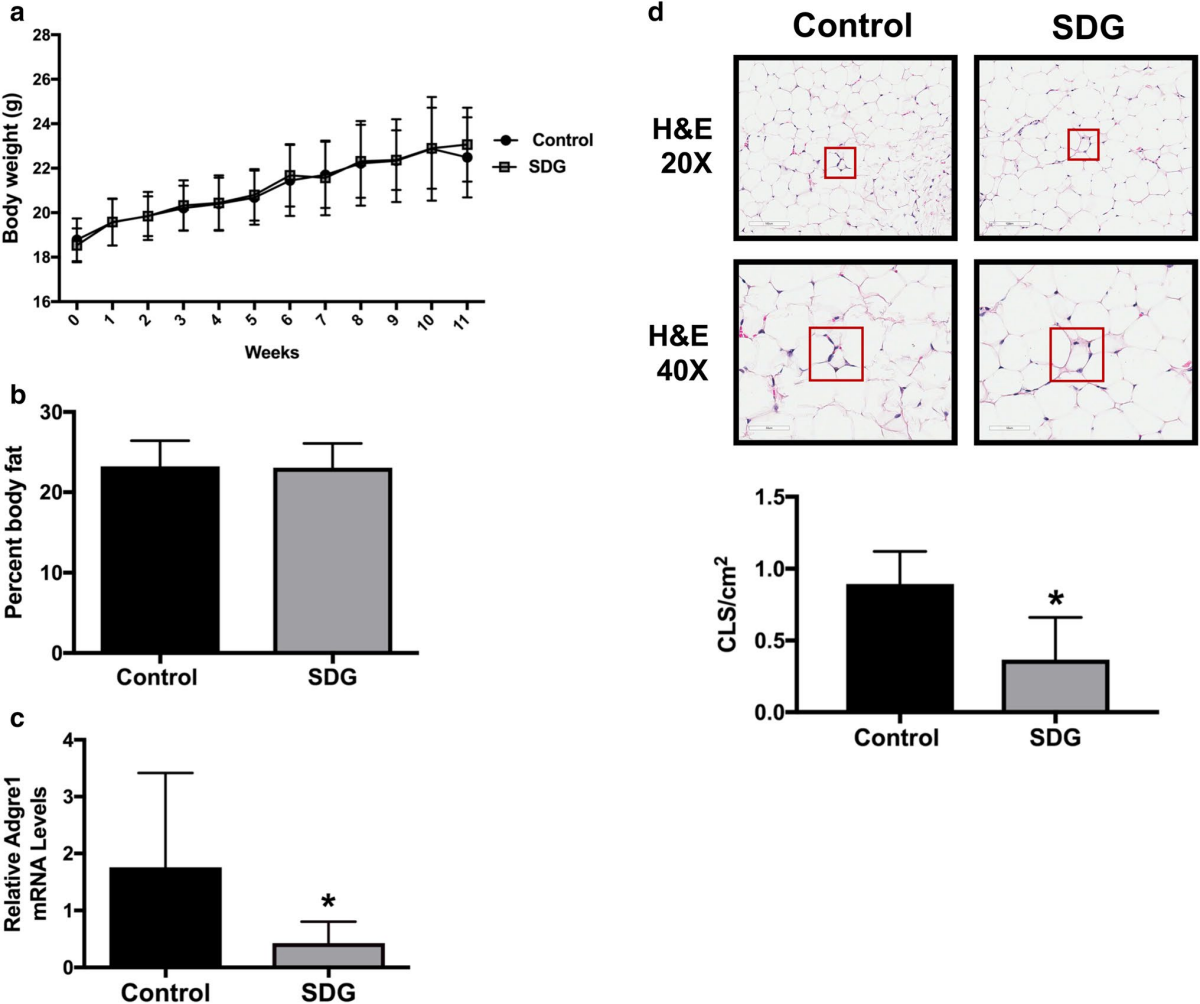
SDG supplementation reduces mammary inflammation. **a** Body weights were measured each week in all mice; mean (± SD) weekly body weights for the mice maintained on the control or SDG-supplemented (SDG) diets are shown. **b** Final percent body fat levels in mice fed the control and SDG diets were measured following euthanization. **c***Adgre1* expression in the 9th (tumor-distal) mammary gland was measured by quantitative RT-PCR in control and SDG-supplemented mice. **d** Prevalence of crown-like structures (CLS) was assessed in the 9th mammary gland of control and SDG-supplemented mice using hematoxylin and eosin (H&E)-stained tissue sections. Representative images shown at × 20 and × 40 magnification. CLS were quantified for each tissue sample as number of CLS per cm^2^. *P < 0.05

No differences were observed in the in vivo tumor study in body weight throughout the 11-week study period or body fat percentage at study termination between control mice versus SDG-supplemented mice (Fig. 2a, b). Given that our pilot study suggested that SDG may have anti-inflammatory effects in the mammary tissue, we next examined whether SDG affected inflammatory markers in the nontumor-bearing mammary gland. Expression of *Adgre1* (the gene for F4/80) and the prevalence of crown-like structures (CLS) were both significantly reduced in the mammary gland of SDG-supplemented mice relative to control mice (*P* < 0.05 for both) (Fig. 2c, d). We also examined SDG’s effects on systemic inflammation by measuring serum levels of 7 inflammatory cytokines (IL-1β, IL-6, IL-10, GM-CSF, IFN-γ, MCP-1, and TNF-α), but found no differences between groups (Online Resource 1).

### SDG inhibits mammary tumor growth and NF-κB activity

**Fig. 3:**
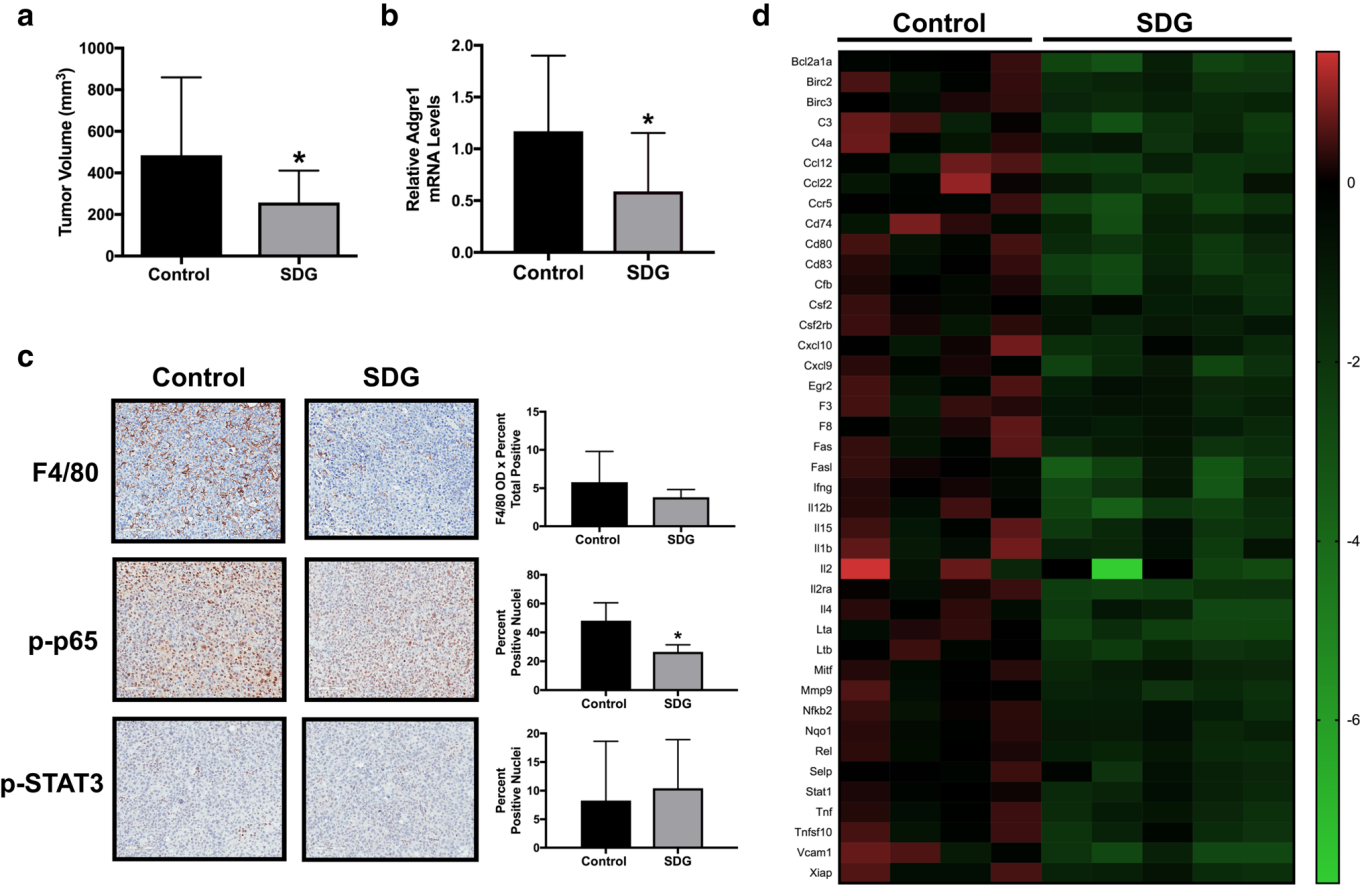
E0771 mammary tumor growth and NF-κB activity are inhibited by SDG supplementation. **a** Final tumor volume in mice fed the control or SDG-supplemented (SDG) diets was measured at necropsy. **b** Tumor *Adgre1* expression was measured by quantitative RT-PCR in control and SDG-supplemented mice. **c** Immunohistochemical staining for tumor F4/80, p-p65 (Ser276), and p-STAT3 (Tyr705) expression in control and SDG-supplemented mice. Representative images shown at x20 magnification. **d** NF-κB target gene expression in tumors from control and SDG-supplemented mice was assessed using a Mouse NF-κB Signaling Target PCR Array; relative expression of genes that were significantly downregulated (*P* < 0.05) by at least 50% in SDG-supplemented mice compared with control mice is displayed in the heat map. *P < 0.05

Final tumor volume was significantly smaller in the mice receiving SDG compared with control mice (*P* < 0.05) (Fig. 3a). Tumor mRNA levels of *Adgre1* were also lower in SDG-supplemented versus control mice (*P* < 0.05) (Fig. 3b). However, F4/80 protein expression was not significantly lower in the tumors from SDG-supplemented mice relative to controls (Fig. 3c). We then assessed the activation of two pathways that mediate the effects of several inflammatory signaling molecules. Tumor expression of p-p65 (Ser276), the activated form of a subunit of the pro-inflammatory transcription factor NFκB, was significantly lower in SDG-supplemented mice compared with controls (*P* < 0.05). In contrast, tumor expression of p-STAT3 (Tyr705) did not differ between SDG-supplemented and control mice (Fig. 3c). We then utilized a Mouse NF-κB Signaling Target PCR Array to examine the impact of SDG on tumor NFκB target gene expression. Out of 84 genes analyzed, the expression of 41 genes was significantly downregulated by at least 50% in SDG-supplemented mice compared with controls (*P* < 0.05), while no genes were significantly upregulated (Fig. 3d).

### ENL decreases breast cancer cell viability and survival in vitro

**Fig. 4:**
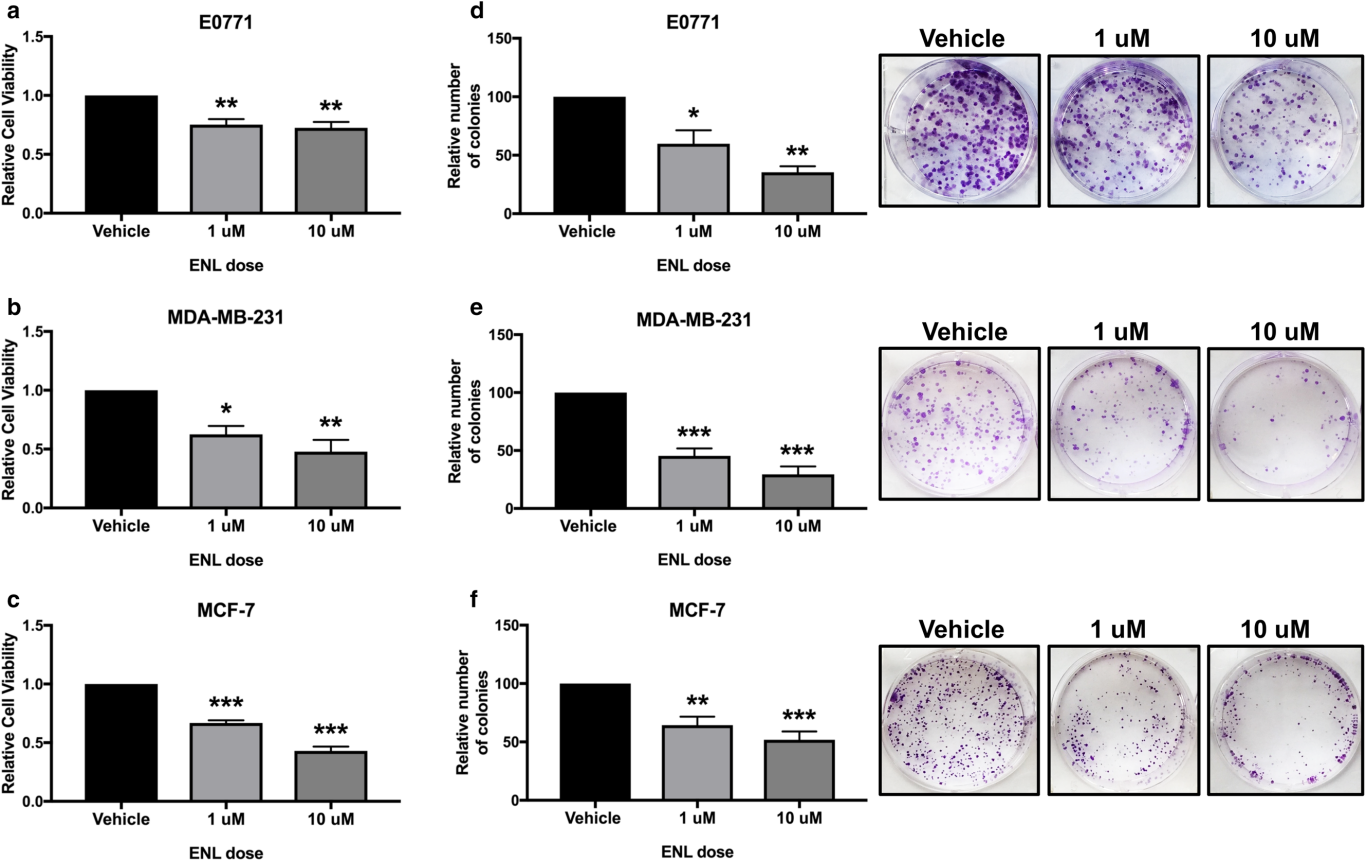
Breast cancer cell viability and survival in vitro are decreased by ENL. **a** E0771, **b** MDA-MB-231, and **c** MCF-7 cell viability following a 48-h treatment with 1 µM or 10 µM ENL was measured by MTT assay. The effects of a 7-day treatment with 1 µM or 10 µM ENL on **d** E0771, **e** MDA-MB-231, and **f** MCF-7 cell survival was quantified by counting the relative number of colonies formed. Representative images were captured by a digital camera. *P < 0.05, **P < 0.01, ***P < 0.001 in comparison with vehicle

To further explore the mechanisms underlying the anticancer effects of SDG, we utilized 3 mammary tumor cell lines, including the same triple-negative E0771 mouse mammary tumor cells used in the tumor study. In addition, a human TNBC cell line, MDA-MB-231, was used to establish whether results seen in the E0771 cells extend to other TNBC cells. Finally, human MCF-7 cells, which model the ERα-positive luminal A subtype of human breast cancer, were used to examine whether any identified mechanisms also mediate ENL’s effects on this disease subtype. Both 1 µM and 10 µM doses of ENL, in comparison with vehicle, significantly decreased cell viability in the E0771 (*P* < 0.01 for both), MDA-MB-231 (1 µM, *P* < 0.05; 10 µM, *P* < 0.01), and MCF-7 (*P* < 0.001 for both) cell lines (Fig. 4a–c). Cell survival was also significantly decreased, relative to vehicle, by both 1 µM and 10 µM ENL in E0771 (1 µM, *P* < 0.05; 10 µM, *P* < 0.01), MDA-MB-231 (*P* < 0.001 for both) and MCF-7 (1 µM, *P* < 0.01; 10 µM, *P* < 0.001) cells (Fig. 4d-f).

### ENL inhibits breast cancer cell NF-κB activity

**Fig. 5:**
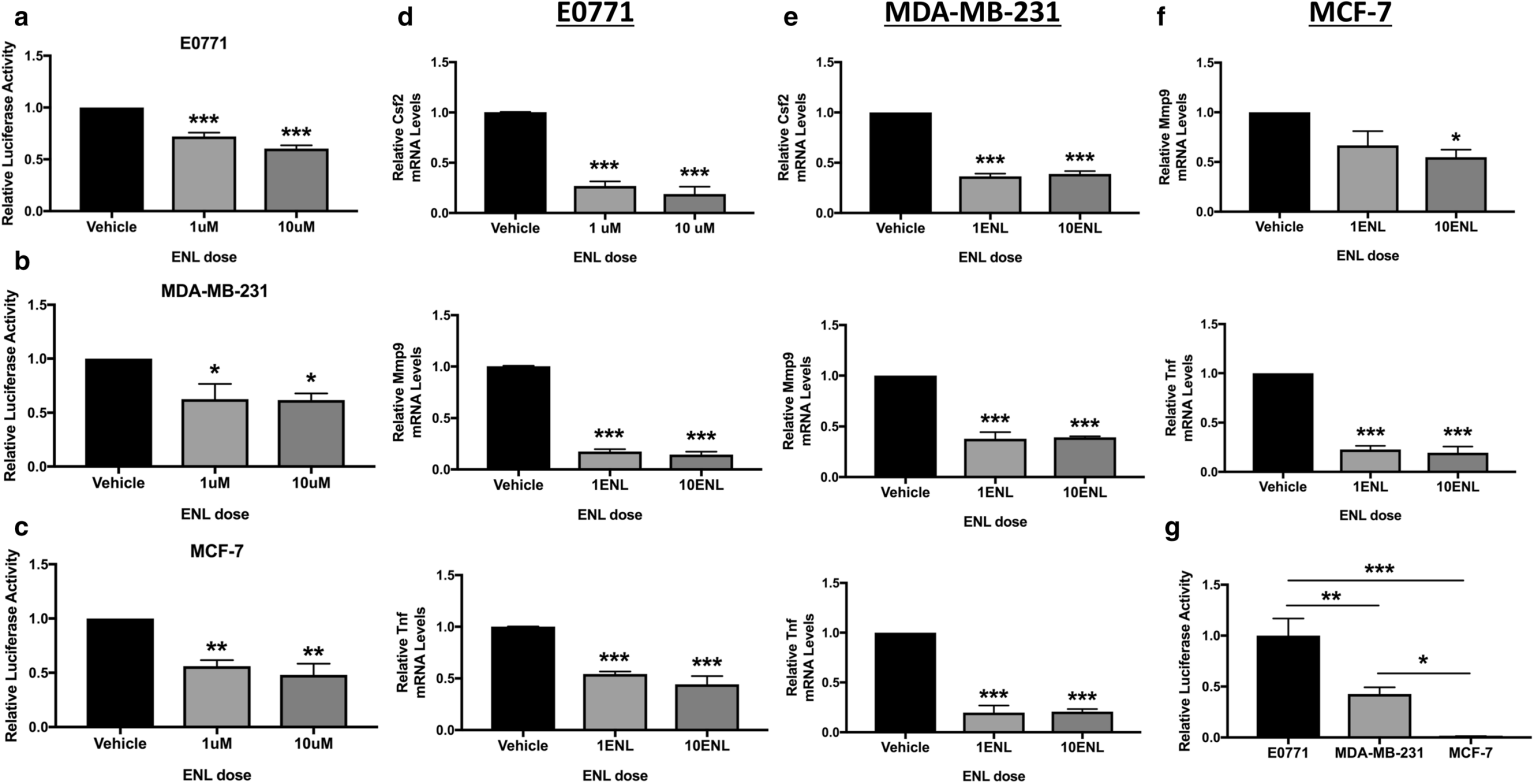
ENL decreases breast cancer cell NF-κB activity. Relative NF-κB luciferase activity was measured in **a** E0771, **b** MDA-MB-231, and **c** MCF-7 cells following a 48-h pretreatment with 1 µM or 10 µM ENL, then a 24-h treatment with 1 µM or 10 µM ENL + lipopolysaccharide (LPS). Expression of NF-κB target genes in **d** E0771, **e** MDA-MB-231, and **f** MCF-7 cells under the same conditions was measured by quantitative RT-PCR. **g** Relative NF-κB luciferase activity in the 3 cell lines under control vehicle conditions was directly compared. *P < 0.05, **P < 0.01, ***P < 0.001 in comparison with vehicle except where a different comparison is indicated by horizontal line

We next examined the impact of ENL on NF-κB activity in the 3 mammary tumor cell lines. Both doses of ENL, in comparison with vehicle, significantly decreased relative luciferase activity in E0771 (*P* < 0.001 for both), MDA-MB-231 (*P* < 0.05 for both), and MCF-7 (*P* < 0.01 for both) cells transfected with an NF-κB reporter (Fig. 5a–c). We then assessed the effects of ENL treatment on 4 NF-κB target genes: *Csf2, Fasl, Mmp9*, and *Tnf*. In E0771 and MDA-MB-231 cells, the expression of *Csf2, Mmp9*, and *Tnf* was significantly decreased by both ENL doses (*P* < 0.001 for all) (Fig. 5d, e). In MCF-7 cells, the expression of *Mmp9* was reduced by 10 µM ENL (*P* < 0.05), while *Tnf* expression was decreased by both ENL doses (*P* < 0.001 for both) (Fig. 5f). *Fasl* expression was not significantly affected by ENL treatment in E0771 or MDA-MB-231 cells (data not shown), and *Csf2* and *Fasl* expression was not detectable in MCF-7 cells. Finally, we found that NF-κB activity in MCF-7 cells was approximately 100-fold and 50-fold lower compared with E0771 cells (*P* < 0.001) and MDA-MB-231 cells (*P* < 0.05), respectively, when measured by dual luciferase assay under vehicle control conditions (Fig. 5g).

### ENL-induced decreases in E0771 cell viability and survival are mediated by inhibition of NF-κB activity

**Fig. 6:**
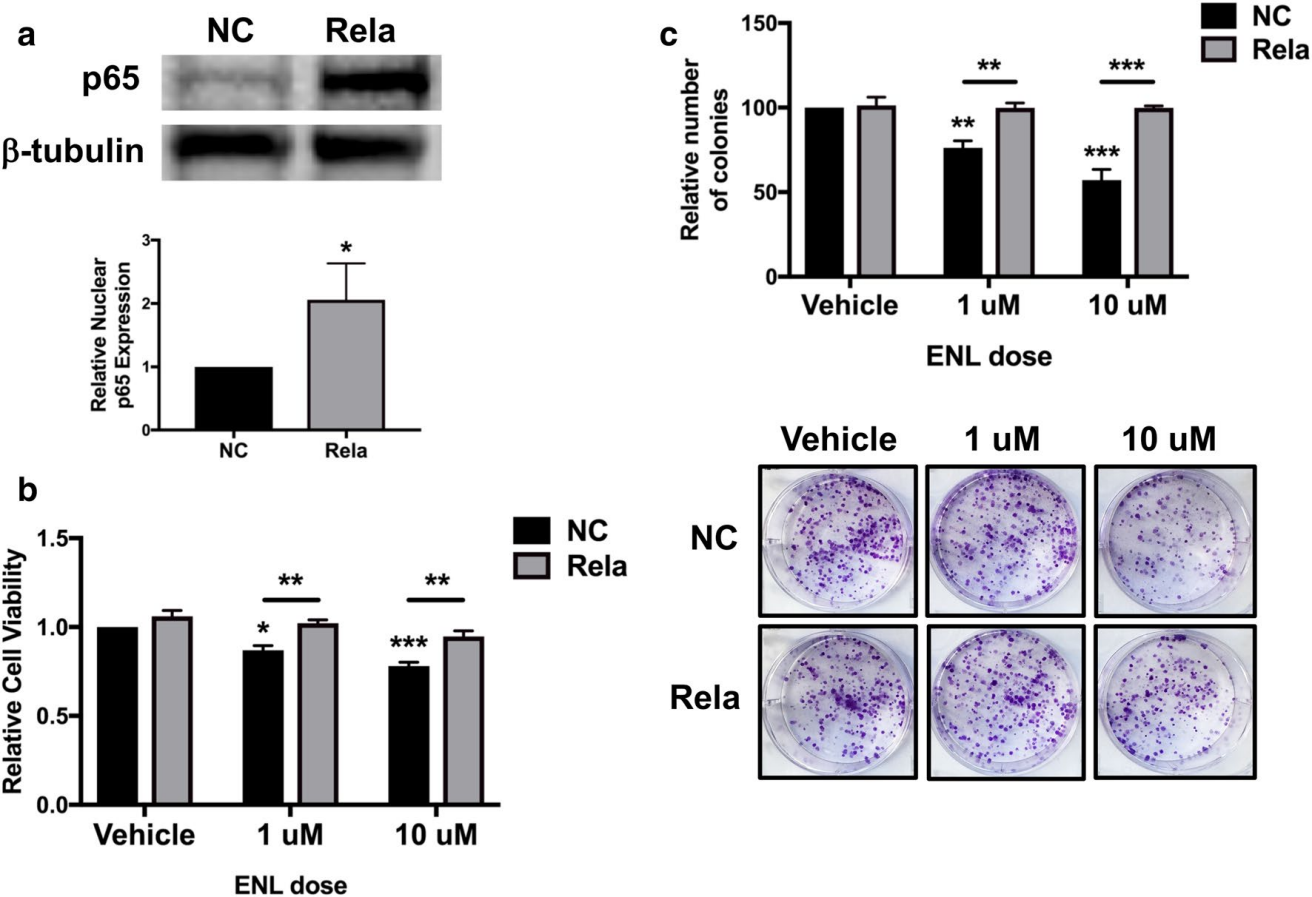
ENL decreases E0771 cell viability and survival via inhibition of NF-κB activity. **a** Nuclear p65 expression in E0771 cells transfected with a negative control vector (NC) or a *Rela* overexpression plasmid (Rela) was measured by western blot analysis. **b** Cell viability was measured by MTT assay in NC- and Rela-transfected E0771 cells following a 48-h treatment with 1 µM or 10 µM ENL. **c** Colony formation was quantified in NC- and Rela-transfected E0771 cells following a 7-day treatment with 1 µM or 10 µM ENL. Representative images were captured by a digital camera. *P < 0.05; **P < 0.01, ***P < 0.001 in comparison with vehicle except where a different comparison is indicated by horizontal line

To determine whether the observed decrease in cell viability and survival following ENL treatment is mediated by an inhibition of NF-κB activity, we transiently transfected E0771 cells with a *Rela* overexpression plasmid (Rela) to induce constitutive overexpression of p65. In comparison with E0771 cells transfected with a negative control vector (NC), nuclear p65 expression was significantly increased (*P* < 0.05) in cells transfected with the Rela plasmid (Fig. 6a). Cell viability and survival were then measured in NC and Rela E0771 cells treated with ENL. In NC cells, both doses of ENL promoted a significant decrease in viability (1 µM, *P* < 0.05; 10 µM, *P* < 0.001), while ENL did not significantly affect the viability of Rela cells (Fig. 6b). *Rela* overexpression also prevented ENL’s effects on E0771 cell survival, as both 1 µM (*P* < 0.01) and 10 µM (*P* < 0.0001) doses significantly reduced the relative number of colonies formed by NC cells, but not Rela cells (Fig. 6c).

## Discussion

While the anti-tumor effects of the flaxseed lignan SDG have been thoroughly established in several models of ERα-positive breast cancer [9–12, 14–17, 35], less attention has been given to its impact on ERα-negative models, including models of basal-like and other triple-negative breast cancer subtypes, and the precise mechanisms mediating their effects. Our findings suggest that SDG may inhibit basal-like breast tumor progression via modulation of NF-κB activity. We specifically report that: (a) SDG supplementation in a mouse model of premenopausal basal-like breast cancer reduces tumor growth and NF-κB activity; (b) in vitro treatment with ENL, the primary bioactive metabolite of SDG, inhibits cell viability, survival, and NF-κB activity in models of basal-like, claudin-low, and luminal A breast cancer; and (c) ENL inhibits viability and survival via modulation of NF-κB activity in the E0771 basal-like breast cancer model, the same model in which SDG inhibited in vivo tumor growth. To our knowledge, this is the first study to identify reduced NF-κB activity as a mediator of ENL’s anti-tumor effects.

NF-κB is a transcription factor that is activated by numerous stimuli, such as growth factors and pro-inflammatory cytokines and chemokines. Its activation increases the expression of genes associated with tumor progression, including genes that promote resistance to apoptotic signals, cell proliferation and survival, angiogenesis, metastasis, and inflammation [36]. Elevated NF-κB activity has been found in many cancers [37] and is particularly high in ERα-negative breast cancer [38–40]. Using various breast cancer models, researchers have previously demonstrated a downregulation in NF-κB activity following treatment with other phytoestrogens, including genistein, daidzein, and quercetin [41–43]. In the current study, we demonstrated a significant decrease in phosphorylated (Ser276) p65, an NF-κB family member, as well as NF-κB target gene expression in the tumors of SDG-supplemented mice. No NF-κB target genes were significantly increased in the SDG-fed mice. This is consistent with a reduction in p-p65 (Ser276), as this phosphorylation increases p65 transcriptional activity [44]. Others have shown that reduced mammary stroma IL-1β production and increased mammary tumor IL-1Ra levels play roles in ENL’s anti-angiogenic effects [10], suggesting that ENL has additional anti-inflammatory effects that contribute to its anti-tumor activity. Furthermore, Jaskulski et al. [45] recently reported that the inverse association between serum ENL and breast cancer-specific mortality is partially mediated by C-reactive protein, providing additional support for the hypothesis that ENL has anti-inflammatory activity. We found that constitutive *Rela* overexpression significantly attenuated ENL’s inhibitory effects on viability and survival in the E0771 cell line, a model of basal-like breast cancer that is considered functionally triple-negative [21, 26, 46, 47], indicating that decreased activation of the p65 subunit of NF-κB is a key mediator of ENL’s effects in these cells. Given that SDG supplementation in the in vivo tumor study significantly decreased E0771 tumor growth and expression of several pro-proliferative and anti-apoptotic NF-κB target genes, including *Bcl2a1a, Birc2, Birc3, Egfr2*, and *Xiap*, we were surprised to find that the 10 µM ENL treatment in vitro inhibited E0771 cell viability by only 27%. This small effect size may be due to the limitations of our cell culture model, which only captured the direct effects of ENL exposure on the cancer cells and did not consider the possible role of other cell types in the tumor microenvironment. However, the same 10 µM ENL treatment reduced E0771 colony formation by 65%, suggesting that ENL does have a direct, biologically relevant impact on E0771 cell survival.

We also demonstrated that ENL reduces cell viability, survival, and NF-κB activity in MDA-MB-231 and MCF-7 cells, models of triple-negative claudin-low and ERα-positive luminal A breast cancer, respectively. Intriguingly, ENL treatment produced a twofold greater inhibition of viability in these 2 cell lines in comparison with the E0771 cells. However, there were no clear differences between the cell lines in inhibition of cell survival or NF-κB activity, so the reason for the difference in viability remains unclear. We also observed that NF-κB activity in the MCF-7 cell line was significantly lower in comparison with the E0771 and MDA-MB-231 cells under vehicle conditions, and 1/2 of the NF-κB target genes assessed were undetectable in this cell line. Consequently, the reduction in NF-κB activity observed in the MCF-7 cell line may not be biologically relevant, and ENL may act to reduce viability and survival in this cell line via an alternate mechanism. Our findings thus suggest that while ENL inhibits cell viability and survival across multiple breast cancer subtypes, biologically relevant ENL-induced inhibition of NF-κB activity may be limited to nonluminal breast cancers, perhaps specifically to the basal-like and claudin-low subtypes.

A small number of prior studies have investigated SDG’s effects in TNBC models. Treatment with ENL and END in vitro has been shown to reduce MDA-MB-231 cell proliferation, adhesion, migration, and invasion [22, 23]. Researchers have also demonstrated that ENL is a radiosensitizer [24] and enhances the cytotoxicity of chemotherapeutic agents [25] in MDA-MB-231 cells. In addition, supplementation with flaxseed oil or SDG in vivo was shown to inhibit metastasis from ERα-negative MDA-MB-435 xenografts [48, 49]. However, MDA-MB-435 cells are now known to be a melanoma cell line based on gene expression profiling and other analyses, so findings from studies utilizing MDA-MB-435 cells should be interpreted with caution.

Our current study examined the impact of ENL on cell lines representing multiple breast cancer subtypes, allowing us to delineate differences between the subtypes. However, there were several limitations to our studies. First, the in vivo studies did not use blinded outcome assessment, which can protect against potential bias in measurements and data reporting. In addition, the in vitro experiments that utilized the *Rela* overexpression plasmid to mechanistically link modulation of NF-κB activity to ENL’s anticancer effects were only performed in the E0771 cell line. Consequently, while MDA-MB-231 cells also responded to ENL with a decrease in NF-κB activity, we cannot conclude that this decrease is the cause of ENL’s effects on cell viability and survival in this cell line. We also cannot generalize our findings regarding ENL’s effects on the MCF-7 cell line to all ERα-positive breast cancer. Given that this study aimed to focus on ERα-negative disease, further exploration of ENL’s effects on ERα-positive cell lines was beyond its scope. Finally, our cell culture experiments did not include examination of the effects of END, the second SDG metabolite. We limited our in vitro investigation to ENL because it is considered the primary SDG enterolignan.

Our in vitro model was also limited by its focus on the direct effects of ENL on cancer cells, without an assessment of how ENL may be affecting the other cell types found in the tumor microenvironment, including macrophages. In the in vivo tumor study, we found a significant decrease in markers of macrophage infiltration in the normal mammary gland of SDG-supplemented mice. Given that inflammation within the tissue microenvironment has been clearly linked to the risk and progression of many types of cancer [50], this decrease in macrophage infiltration may play a role in SDG’s anti-tumor effects. However, we did not explore this factor in our in vitro model, and it should thus be addressed in future studies. We also found that mice fed the SDG diet had lower tumor expression of *Adgre1*, the gene for the macrophage marker F4/80, but F4/80 protein expression was not significantly reduced in the SDG-supplemented mice. Consequently, we cannot conclude that tumor macrophage infiltration is affected by SDG in this model.

In summary, we have demonstrated that SDG reduces tumor growth in the E0771 model of basal-like breast cancer, likely via a mechanism involving inhibition of NF-κB activity. SDG has a highly favorable safety profile [27], and there is substantial evidence from population studies linking greater lignan exposure to reduced breast cancer mortality, including for ERα-negative disease [4–8, 51, 52]. Consequently, SDG could serve as a practical and effective adjuvant treatment for the prevention of recurrence.

## Electronic supplementary material

Below is the link to the electronic supplementary material.

Supplementary material 1 (DOCX 14 KB)

## Data Availability

The datasets used and/or analyzed during the current study are available from the corresponding author on reasonable request.

## Abbreviations

ANOVA: Analysis of variance
CLS: Crown-like structure
END: Enterodiol
ENL: Enterolactone
ERα: Estrogen receptor alpha
GM-CSF: Granulocyte–macrophage colony-stimulating factor
IFN-γ: Interferon gamma
IL-1β: Interleukin 1 beta
IL-6: Interleukin 6
IL-10: Interleukin 10
LPS: Lipopolysaccharide
MCP-1: Macrophage chemoattractant protein 1
NC: Negative control vector
NF-κB: Nuclear factor-kappa B
SD: Standard deviation
SDG: Secoisolariciresinol diglucoside
SEM: Standard error of the mean
STAT3: Signal transducer and activator of transcription 3
TNBC: Triple-negative breast cancer
TNF-α: Tumor necrosis factor alpha
