# Supplementary material for: The flaxseed lignan secoisolariciresinol diglucoside decreases local inflammation, suppresses NFκB signaling, and inhibits mammary tumor growth
Source: Breast Cancer Res Treat. 2018 Oct 26;173(3):545–57. doi: 10.1007/s10549-018-5021-6 (PMC6394576; doi:10.1007/s10549-018-5021-6)
Supplement: Supplementary file 1 — Supplementary material 1 (DOCX 14 KB) [file 10549_2018_5021_MOESM1_ESM.docx]

**The flaxseed lignan secoisolariciresinol diglycoside decreases local inflammation, suppresses NFκB signaling, and inhibits mammary tumor growth**

*Breast Cancer Research and Treatment*

Laura W. Bowers, Claire G. Lineberger, Nikki A. Ford, Emily L. Rossi, Arunima Punjala, Bruce K. Kimler, Carol J. Fabian, Stephen D. Hursting

Corresponding author: Stephen D. Hursting, PhD, MPH; Department of Nutrition, University of North Carolina at Chapel Hill; hursting@email.unc.edu

**Supplementary Table 1.** Serum cytokines in C57BL/6 mice maintained on control or SDG-supplemented diet regimens

**Analyte Control SDG p-value**

IL-1β (ng/mL) 0.30 (0.08) 0.27 (0.07) 0.43

IL-6 (pg/mL) 10.8 (4.2) 11.9 (3.2) 0.54

IL-10 (ng/mL) 0.13 (0.03) 0.13 (0.04) 0.76

GM-CSF (ng/mL) 0.12 (0.03) 0.13 (0.01) 0.81

IFN-γ (pg/mL) 39.5 (13) 43.7 (8.9) 0.42

MCP-1 (ng/mL) 0.30 (0.07) 0.29 (0.05) 0.86

TNF-α (ng/mL) 1.46 (0.55) 1.62 (0.25) 0.41

Multiplex immunoassay analyses performed on serum collected at euthanization (n=10/group). Standard deviations shown in parentheses. Abbreviations: IL-1β, interleukin 1 beta; IL-6, interleukin 6; IL-10, interleukin 10; GM-CSF, granulocyte-macrophage colony-stimulating factor; IFN-γ, interferon gamma; MCP-1, macrophage chemoattractant protein 1; TNF-α, tumor necrosis factor alpha.
